# Factors associated with self-perceived health status in Portugal: Results from the National Health Survey 2014

**DOI:** 10.3389/fpubh.2022.879432

**Published:** 2022-09-06

**Authors:** Ahmed Nabil Shaaban, Maria Rosario O. Martins, Bárbara Peleteiro

**Affiliations:** ^1^Department of Global Public Health, Karolinska Institutet, Solna, Sweden; ^2^Global Health and Tropical Medicine, Institute of Hygiene and Tropical Medicine, NOVA University of Lisbon, Lisbon, Portugal; ^3^Epidemiology Research Unit (EPIUnit) – Instituto de Saúde Pública, Universidade do Porto, Porto, Portugal; ^4^Departamento de Ciências da Saúde Pública e Forenses e Educação Médica Faculdade de Medicina da Universidade do Porto, Porto, Portugal

**Keywords:** self-perceived health status, socioeconomic status, social isolation, barriers to access, mental health, health inequalities

## Abstract

**Background:**

Self-perceived health is an important indicator of illness and mortality. This study aims at identifying a wide range of factors that can influence self-perceived health status among a representative sample in Portugal.

**Methods:**

We used the 2014 National Health Survey (*n* = 17,057), whereby participants were required to assess their health status from “Very good,” “Good,” “Fair,” “Poor” to “Very poor.” We grouped the answers “Very good” and “Good,” and “Poor” and “Very poor,” respectively. Multinomial logistic regression was used to compare participants' characteristics across groups by computing odds ratio and corresponding 95% confidence intervals. Models included Socioeconomic/demographic characteristics, objective health status, healthcare use, functional disability, barriers to healthcare services utilization, lifestyle variables, mental health status, social support, and satisfaction with life as potential factors that can affect self-perceived health. Models were adjusted for sex, age, educational level, degree of urbanization, and presence of chronic diseases.

**Results:**

About 45% of participants reported good/very good, 39% reported fair, while ~16% reported poor/very poor health perception. Poor/very poor health was more reported by women when compared to men (19.1 vs. 11.4%, respectively, *p* < 0.001). A higher prevalence of poor/very poor health status was reported by participants living in thinly populated areas or among older populations. Lower educational levels, lower income, as well as unemployment, were found to increase the risk of reporting poor/very poor health status. Utilizing healthcare services more frequently, experiencing barriers to access healthcare services, having depressive symptoms or activity limitations, or lacking social support were found to be significantly associated with poor/very poor self-perceived health.

**Conclusion:**

Subjects living in Portugal tend to report less good/very good health status and more poor/very poor health when compared to the rest of Europe. This study stresses the importance of socioeconomic factors, chronic illness, barriers to access healthcare services, social isolation, and mental health status in influencing self-perceived health and highlights the urgent need for social-informed policies, strategies, and interventions to reduce health inequalities in Portugal.

## Background

An enormous amount of research has examined self-perceived health status due to its capability to summarize more objective measures, namely morbidity, mortality, and clinical assessments of health conditions (1, 2). Given this importance, scholars have conducted several studies on risk factors and policy interventions that can impact self-perceived health by employing self-perceived health as a health outcome (3–5).

Portugal is recording one of the highest rates of poor perceived health in Europe (6); it is, therefore, crucial to define the factors that can affect self-perceived health status in Portugal and to which degree it may translate into real health inequalities. Previous studies in Portugal that assessed predictors of self-perceived health status used data limited to specific geographic areas (7, 8), population groups, such as adolescents (9, 10), elderly (11), or patients with morbidities (12, 13), or, if using national data, limited the analysis to socio-demographics and chronic diseases (14). However, there is evidence that other factors may play an important role in shaping self-perceived health, such as mental health (15, 16), social support (17, 18), satisfaction with life (16, 19), healthcare use (20), functional ability (21, 22) and lifestyle factors (23, 24). Ignoring these factors in Portugal may undermine their effect on health and, accordingly, lose the potential in providing policy implications for healthcare providers.

Accordingly, the purpose of our study was to identify a wide range of factors that can impact self-perceived health status in Portugal by using data collected at the National Health Survey of 2014. Moreover, this study is aiming to assess to which extent inequalities and disparities affect self-perceived health among individuals included in our study.

## Methods

### Study participants

The present analysis is based on data collected as part of the National Health Survey 2014 (25, 26), which is a community-based cross-sectional study that evaluated a sample of the population living in Portugal (according to NUTS II—Nomenclature of territorial units for statistics, 2 levels), obtained through multistage stratified and cluster sampling.

Using data from the 2011 Population and Housing Census, a sample of households was defined to be the sampling frame for household surveys conducted by Statistics Portugal. This included 1,183 primary sampling units (PSU), selected systematically within larger geographical strata, with a probability proportional to the number of households in each unit. A random sample of the households was then selected, and all persons aged 15 or above living in these households at the date of the recruitment were eligible. In each household, the selected individual was the one whose previous birthday was closest to the date of the contact. The sample size was defined to ensure a homogeneous distribution of the participants by the nine NUTS II regions.

As the National Health Survey uses a multistage, stratified, and cluster sampling, to take into account this study design, sampling weights are used in the analyses. These sampling weights were computed by Statistics Portugal, and are available for each individual in the survey database.

### Data collection

Between September and December 2014, 22,538 households were contacted, and 18,204 persons were evaluated. Information was collected by using either computer-assisted personal interviewing or computer-assisted web interviewing (50% in each stratum). The questionnaire covered four thematic areas: health status, healthcare, health determinants, and income, and health expenses. Self-perceived health status at the time of the interview was collected as part of the health status characterization. We further excluded subjects for whom there was incomplete data on the factors analyzed in this study, resulting in a final sample size of 17,057 subjects.

### Variables

#### Dependent variable

Self-perceived health status at the time of the interview was collected as part of the health status characterization. Participants were asked the question “Overall, how would you rate your health status?,” which was followed by the options “Very good,” “Good,” “Fair,” “Poor” and “Very poor.” The option “Prefers not to answer” was also available, and these participants were excluded (*n* = 9). For analysis, we grouped the answers “Very good” and “Good,” and “Poor” and “Very poor,” respectively. Self-perceived health has proven its ability to summarize objective health outcomes such as morbidity, mortality, and health care utilization (1, 27). Self-perceived health status has been formulated and validated within the Minimum European Health Module (MEHM). The MEHM is a set of three general questions characterizing three different concepts of health that includes self-perceived health (28). The module was developed to be used in all social surveys and is at present implemented in the European Health Interview Survey (EHIS) and EU Statistics on Income and Living Conditions (EU-SILC) (28).

#### Independent variables

The selection of the independent variables was based on the established evidence from previous studies. Accordingly, we included a wide range of variables that may pertain to self-perceived health status, and they fall into seven main categories as follows:

##### Socioeconomic/demographic

Our Socioeconomic/demographic variables include participants' sex (male, female), legal marital status (Single, Married, Divorced, Widowed), Size of household (1, 2, 3, 4, >4), age (categorized into seven categories 15–29, 30–39, 40–49, 50–59, 60–69, 70–79, ≥80), region of residence (Norte Centro Lisboa Alentejo Algarve R.A. Açores R.A. Madeira). Region of residence were classified according to Nomenclature of territorial units for statistics, 2 level (NUTS II) (29). We have also included degree of urbanization (Densely populated area, Intermediate density area, Thinly populated area) based on the share of local population living in urban clusters and urban centers according to the Commission Directorates-General for Regional and Urban Policy, Agriculture and Rural Development (30). Independent variables that measure socioeconomic position included income classified into five categories according to income quintile groups (31) that are computed on the basis of the total equivalized disposable income (32) attributed to each member of the household. We included level of education that measures the highest degree of education according to the International Standard Classification of Education (ISCED), adopted by the United Nations Educational, Scientific and Cultural Organization (UNESCO) (33). For analysis, we grouped the education variables as following (No basic level completed, Second basic level completed, Third basic level completed, Secondary level completed, Higher level completed). We have also included Employment status (Employed, Unemployed, Student, Retired/Disabled, Housewife), and Migration status according to country of birth and nationality (No—born in Portugal and having Portuguese nationality, Yes—born in other countries and having Portuguese nationality, or born in Portugal or other countries and not having Portuguese nationality). The socioeconomic factors have been widely used to assess health in research. For example, better socioeconomic status in terms of higher education, employment, or income may translate to better life conditions, and access to information and hence better health outcomes (34).

##### Objective health status, healthcare use, and functional disability

Factors that measure illness or indicate recent healthcare use or disability, were measured using categorical variables as follows: having ***a*** chronic disease (No, Yes), Consumption of medication with prescription in the last 2 weeks (No, Yes), Consumption of medication without prescription in the last 2 weeks (No, Yes), Hospital admission in the last 12 months (No, Yes), Visits to the hospital for ambulatory health care in the past 12 months (No, Yes). Health care use categorical variables included: Last appointment with general practitioner [more than 1 year ago (includes never), in the last year], last appointment with specialist doctor [more than 1 year ago (includes never), in the last year], last appointment with a dentist [more than 1 year ago (includes never), in the last year], Last appointment with a psychologist, psychotherapist, psychiatrist [more than 1 year ago (includes never), in the last year], Other indicators of healthcare use included Blood pressure measured by a health professional (never or more than 1 year ago, in the last year), cholesterol measured by a health professional (never or more than 1 year ago, in the last year), glycaemia measured by a health professional (never or more than 1 year, In the last year), fecal occult blood test and/or colonoscopy use [never, ever (at least once)], Mammography use e [never, ever (at least once)], Cervical cytology use [never, ever (at least once)]. Variables that measure disability included: General activity limitation (not limited, limited but not severely, severely limited), Absence to work for individual health problems (No, Yes), intensity of pain felt in the previous 4 weeks (No, very slight/slight, moderate, intense/very intense), interference from pain in the usual tasks (nothing, a little/moderately, very/in an extreme way).

##### Barriers to healthcare services utilization

This category included variables that measure health care coverage such as type of public healthcare provider (National health service, National health service, and other subsystems) and having Private health insurance (No, Yes). In addition, variables that measures barriers to access health care included waiting for a consultation, exam, or treatment, in the last 12 months (No, Yes, No need), waiting for a consultation, exam or treatment due to distance and/or transportation in the last 12 months (No, Yes, No need), waiting for a medical consultation, exam or treatment due to financial difficulties in the last 12 months (No, Yes, No need), waiting for a dentist consultation, exam or treatment due to financial difficulties in the last 12 months (No, Yes, No need), waiting for a mental health consultation, exam or treatment due to financial difficulties in the last 12 months (No, Yes, No need), not buying medication due to financial difficulties in the last 12 months (No, Yes, No need). Poor access to health services may exacerbate health conditions, resulting in poor health outcomes (35).

##### Lifestyle variables

We calculated the body mass index (BMI) using the equation BMI = kg/m^2^ where kg is the participant's weight in kilograms and m^2^ is the squared height in meters. We categorized BMI into 4 groups as following (underweight: BMI <18.5 kg/m^2^, normal: 18.5–24.9 kg/m^2^, overweight: 25–29.9 kg/m^2^, and obese: ≥30 kg/m^2^). Other lifestyle factors included: condition on tobacco consumption (Never, Former, Current), drinking status (Never, Former, Current), fruits and vegetables (portions per day) (<5, ≥5). Health can be strongly affected by individual's lifestyle, and the connections between morbidities and several lifestyle factors such as physical inactivity, tobacco consumption, drinking, and eating have been established in several studies (36).

##### Mental health

Mental health at the time of the interview was collected as part of the health status characterization. We assessed the effect of mental health on self-perceived health by including five categorical items, namely, depressed mood frequency in the last 2 weeks (Never, Ever), frequency of sleep disorders in the last 2 weeks (Never, Ever), frequency of fatigue in the last 2 weeks (Never, Ever), frequency of appetite change in the last 2 weeks (Never, Ever), frequency of feeling of uselessness or guilt in the last 2 weeks (Never, Ever), frequency of difficulty concentrating in the last 2 weeks (Never, Ever). A well-established link between mental and physical health has been established elsewhere (37, 38).

##### Social support

We examined the availability of social support using the following categorical variables: number of persons close to the participant whom they could seek in the event of a serious personal problem (6 or more, 3 to 5, 1 to 2, No), level of concern or interest of other people in relation to the participant (Some concern and interest/a lot of concern and interest, Cannot evaluate, No concern and interest/little concern and interest), and degree of perception of getting help from neighbors in case of need (Very easy/easy, Possible, Difficult/very difficult). Social support is strongly associated with lower morbidity and mortality rates compared to socially deprived individuals (39).

##### Satisfaction with life variables

Satisfaction with life is defined as the cognitive evaluation of life as a whole (40). A lower degree of satisfaction in life is a predictor of mortality and morbidity and is linked to poor self-perceived health and unhealthy behaviors (41). However, little is known about the relation between satisfaction with life and health status in Portugal despite this importance. We took the advantage that questions that reflect satisfaction with life questions have been introduced for the first time in the National Health Survey 2014, in which Satisfaction with life at the time of the interview was collected as part of the health determinants. Factors that measures satisfaction with life included: Self-appreciation of proximity to the participant's ideals of life (More or less in agreement/in agreement/totally in agreement, Neither in agreement nor in disagreement, Totally in disagreement/in disagreement/more or less in disagreement), Self-appreciation of the participant's satisfaction with living conditions (More or less in agreement/in agreement/totally in agreement, Neither in agreement nor in disagreement, Totally in disagreement/in disagreement/more or less in disagreement), Self-appreciation of the participant's life satisfaction (More or less in agreement/in agreement/totally in agreement, Neither in agreement nor in disagreement, Totally in disagreement/in disagreement/more or less in disagreement), Self-appreciation of obtaining the most important in life (More or less in agreement/in agreement/totally in agreement, Neither in agreement nor in disagreement, Totally in disagreement/in disagreement/more or less in disagreement), Self-appreciation with the life-path satisfaction (More or less in agreement/in agreement/totally in agreement, Neither in agreement nor in disagreement, Totally in disagreement/in disagreement/more or less in disagreement).

### Statistical analysis

Sociodemographic characteristics across groups were compared using the Pearson chi-squared statistic. Multinomial logistic regression was used to compare participants' characteristics across groups by computing odds ratio (OR) and corresponding 95% confidence intervals (95%CI).

Models included Socioeconomic/demographic characteristics, objective health status, healthcare use, functional disability, barriers to healthcare services utilization, lifestyle variables, mental health status, social support, and satisfaction with life as potential factors that can affect self-perceived health. Models were adjusted for sex, age, educational level, degree of urbanization, and presence of chronic diseases using the participants reporting a “Fair” health status as the reference group. All analyses were conducted with STATA^®^, version 11.2 (StataCorp LP, College Station, Texas, USA), using sampling weights computed based on the design weight, i.e., the inverse of the probability of selection of each PSU and each household within each PSU, further corrected for non-responses and for the effective number of subjects evaluated, regarding the age- and sex-structures.

## Results

Table 1 summarizes the socio-economic/demographic characteristics of the study participants. Our study population was mainly composed of subjects living in densely populated areas (43.2%), married (56.7%), and employed (47.7%), with 8.6% of them having no basic educational level completed, 8.0% of migrants, and more than half reported having a chronic disease (56.4%).

**Table 1:** Characteristics of the study sample (*n* = 17,057).

	***N***	**Weighted %**
**Region of residence (NUTS II)**		
Norte	2,550	34.8
Centro	3,458	22.1
Lisboa	1,856	26.7
Alentejo	2,681	7.4
Algarve	2,396	4.1
R. A. Açores	1,930	2.3
R. A. Madeira	2,186	2.5
**Degree of urbanization^a^**		
Densely populated area	5,066	43.2
Intermediate density area	5,557	29.4
Thinly populated area	6,434	27.4
**Sex**		
Women	7,465	43.8
Men	9,592	56.2
**Age groups (years)**		
15–29	2,021	19.0
30–39	2,513	16.6
40–49	3,005	17.7
50–59	2,855	16.6
60–69	2,912	13.9
70–79	2,376	10.3
≥80	1,375	6.0
**Legal marital status**		
Single	4,204	29.4
Married	8,896	56.7
Divorced	1,618	6.5
Widowed	2,339	7.4
**Educational level**		
No basic level completed	2,077	8.6
Second basic level completed	6,799	34.6
Third basic level completed	2,923	19.6
Secondary level completed	2,769	20.0
Higher level completed	2,489	17.2
**Employment status**		
Employed	7,428	47.7
Unemployed	1962	12.6
Student	915	8.8
Retired/Disabled	5,565	25.1
Housewife	1,187	5.8
**Size of household (including respondent)**		
1	3,853	9.5
2	5,783	28.9
3	3,772	29.2
4	2,678	23.1
>4	971	9.4
**Household income per adult**		
Quintile 1	3,712	19.9
Quintile 2	3,503	19.8
Quintile 3	3,392	20.2
Quintile 4	3,252	20.0
Quintile 5	3,198	20.1
**Migration status^b^**		
No	15,792	92.0
Yes	1,265	8.0
**Chronic disease**		
No	6,633	43.6
Yes	10,424	56.4

NUTS II, Nomenclatura de Unidades Territoriais para Fins Estatísticos, nível II (Nomenclature of territorial units for statistics, 2 level); R.A., Região Autónoma (Autonomous Region).

a Based on the share of local population living in urban clusters and in urban centers according to the Commission Directorates-General for Regional and Urban Policy, Agriculture and Rural Development, Eurostat, Joint Research Center and Organization for Economic Co-operation and Development.

b According to country of birth and nationality (No—born in Portugal and having Portuguese nationality, Yes—born in other countries and having Portuguese nationality, or born in Portugal or in other countries and not having Portuguese nationality).

**Figure 1:**
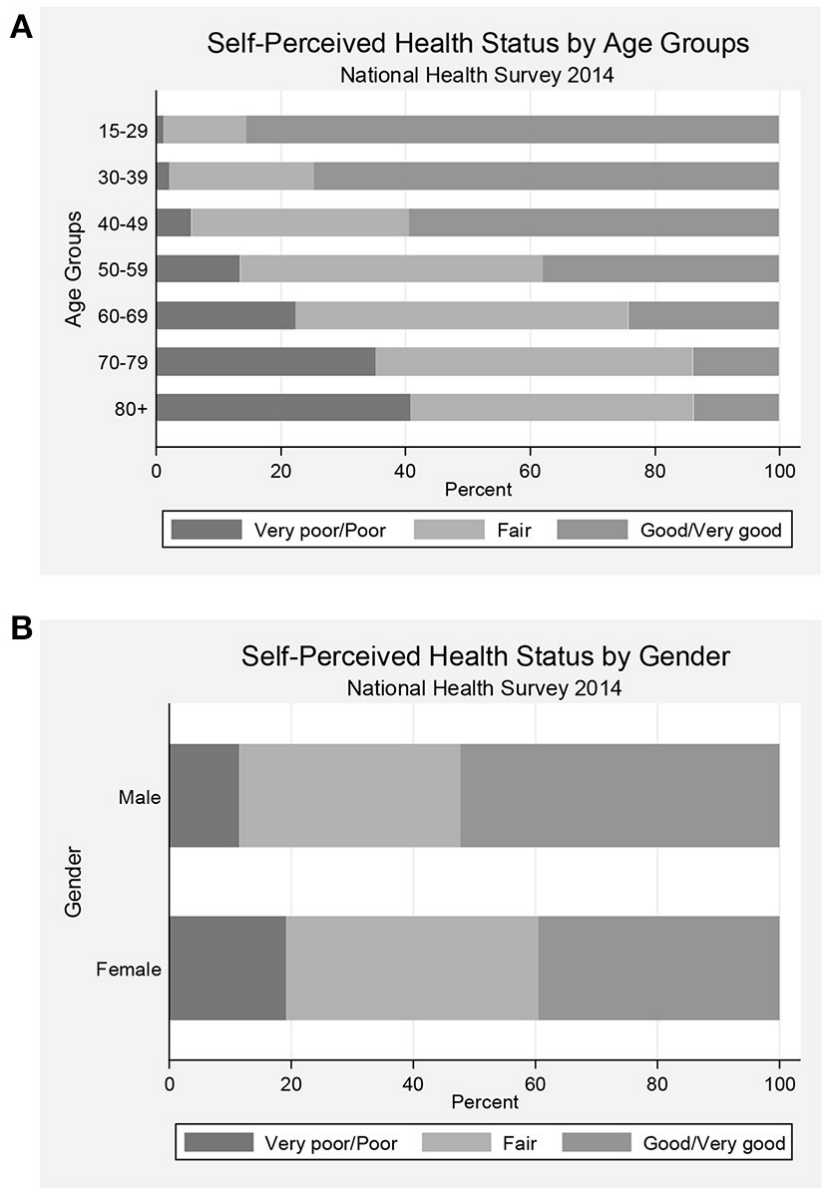
Prevalence of health perception status categories in Portugal according to age group **(A)** and sex **(B)**.

Our results showed that 45.2% of the participants reported good or very good, 39.1% reported fair, while 15.7% reported poor or very poor health perception. There was an increase in the prevalence of very poor/poor self-perceived health status across age groups (from 1.1% in the younger to 40.8% among the older, *p* < 0.001), together with a decrease in the prevalence of good/very good health perception (from 85.5% in the younger to 13.8% among the older, *p* < 0.001) (Figure 1A). In Figure 1B, we observe that women self-rated their health status as poor/very poor in higher frequency than men (19.1 vs. 11.4%, *p* < 0.001), while men had a higher prevalence of good/very good health perception than women (52.3 vs. 39.6%, *p* < 0.001).

Table 2 shows the association between socio-economic/demographic variables and self-perceived health status, using the group of participants classifying their health status as “Fair” as the reference. Participants living in *Alentejo* and *Algarve* were more likely to self-rate their health status as good/very good [adjusted OR (AOR) = 1.24, 95%CI: 1.01–1.51, and AOR = 1.34, 95%CI: 1.12–1.60, respectively], and those living in *Açores* were less likely to report a poor/very poor health status (AOR = 0.79, 95%CI: 0.63–0.99), compared with those living in the *Norte*. A higher prevalence of poor/very poor health status was reported by participants living in thinly populated areas (AOR = 1.52, 95%CI: 1.28–1.80), whereas a lower prevalence of good/very good self-rated health status was observed in these same areas (AOR = 0.75, 95%CI: 0.65–0.87) and intermediate populated areas (AOR = 0.80, 95%CI: 0.69–0.93), compared with those living in densely populated areas. A trend toward a better self-reported health status was observed with increasing educational levels and household income. Compared with employed subjects, individuals that were inactive were more likely to self-rate their health status as poor/very poor. There were no statistically significant differences in self-perceived health status according to legal marital status, size of the household, and migration status.

**Table 2:** Association between socio-economic characteristics and self-perceived health status.

	**Self-perceived health status**						
	**Poor/very poor**			**Fair**	**Good/very good**		
	**Weighted %**	**Crude OR (95%CI)**	**Adjusted^a^ OR (95%CI)**	**Weighted %**	**Weighted %**	**Crude OR (95%CI)**	**Adjusted^a^ OR (95%CI)**
**Region of residence (NUTS II)**							
Norte	36.6	1 [reference]	1 [reference]	35.3	34.0	1 [reference]	1 [reference]
Centro	26.9	1.11 (0.94–1.32)	0.96 (0.79–1.17)	23.4	20.1	0.89 (0.78–1.01)	0.96 (0.81–1.14)
Lisboa	19.4	0.75 (0.61–0.93)	0.86 (0.67–1.10)	24.9	20.8	1.24 (1.07–1.43)	1.08 (0.89–1.30)
Alentejo	9.3	1.21(1.02–1.42)	0.85 (0.69–1.05)	7.4	6.9	0.98 (0.85–1.12)	1.24 (1.01–1.51)
Algarve	4.1	0.99 (0.83–1.20)	0.89 (0.73–1.08)	4.0	4.3	1.12 (0.98–1.29)	1.34 (1.12–1.60)
R.A. Açores	1.8	0.79 (0.63–0.97)	0.79 (0.63–0.99)	2.2	2.5	1.15 (0.99–1.33)	1.07 (0.88–1.31)
R.A. Madeira	1.9	0.64 (0.52–0.78)	0.64 (0.52–0.79)	2.8	2.4	0.88 (0.77–1.01)	0.79 (0.66–0.94)
**Degree of urbanization^b^**							
Densely populated area	32.6	1 [reference]	1 [reference]	41.1	47.3	1 [reference]	1 [reference]
Intermediate density area	27.0	1.15 (0.96–1.38)	1.18 (0.98–1.42)	29.6	29.9	0.88 (0.78–0.99)	0.80 (0.69–0.93)
Thinly populated area	40.4	1.74 (1.48–2.04)	1.52 (1.28–1.80)	29.3	22.8	0.68 (0.60–0.76)	0.75 (0.65–0.87)
**Sex**							
Men	64.5	1 [reference]	1 [reference]	42.7	35.5	1 [reference]	1 [reference]
Women	35.5	1.35 (1.18–1.56)	1.21 (1.04–1.40)	57.3	64.5	0.67 (0.60–0.74)	0.62 (0.55–0.70)
**Age groups (years)**							
15–29	1.6	1 [reference]	1 [reference]	6.6	31.7	1 [reference]	1 [reference]
30–39	2.9	1.17 (0.54–2.55)	1.14 (0.51–2.52)	10.1	24.5	0.50 (0.40–0.64)	0.47 (0.37–0.61)
40–49	7.9	1.85 (0.91–3.76)	1.47 (0.72–2.99)	17.5	20.3	0.24 (0.19–0.30)	0.29 (0.23–0.37)
50–59	18.2	3.38 (1.70–6.73)	2.10 (1.05–4.20)	22.0	12.5	0.12 (0.09–0.15)	0.19 (0.15–0.25)
60–69	23.3	4.50(2.28–8.89)	2.43 (1.22–4.84)	21.2	6.6	0.06 (0.05–0.08)	0.13 (0.10–0.17)
70–79	27.8	7.56 (3.83–14.93)	3.31 (1.61–6.41)	15.1	2.7	0.04 (0.03–0.05)	0.09 (0.07–0.13)
≥80	18.3	9.98 (5.00–19.91)	3.76 (1.86–7.60)	7.5	1.8	0.05 (0.04–0.07)	0.15 (0.10–0.21)
**Legal marital status**							
Single	9.0	1 [reference]	1 [reference]	15.7	43.8	1 [reference]	1 [reference]
Married	66.8	1.74 (1.36–2.22)	1.01 (0.76–1.36)	66.9	47.3	0.25 (0.22–0.29)	0.83 (0.68–1.02)
Divorced	5.1	1.26 (0.90–1.78)	1.07 (0.73–1.58)	7.0	6.6	0.34 (0.28–0.41)	0.90 (0.69–1.17)
Widowed	19.1	3.18 (2.42–4.17)	0.89 (0.64–1.23)	10.5	2.4	0.08 (0.07–0.10)	0.81 (0.60–1.10)
**Educational level**							1 [reference]
No basic level completed	29.8	1 [reference]	1 [reference]	10.6	2.0	1 [reference]	
Second basic level completed	54.1	0.30 (0.33–0.46)	0.57 (0.47–0.69)	49.4	19.7	2.15 (1.70–2.72)	1.07 (0.82–1.40)
Third basic level completed	8.5	0.18 (0.13–0.23)	0.37 (0.27–0.49)	17.1	24.1	7.60 (5.94–9.72)	1.85 (1.37–2.50)
Secondary level completed	4.5	0.13 (0.09–0.18)	0.31 (0.22–0.44)	13.4	28.4	11.45 (8.90–14.74)	2.80 (2.07–3.79)
Higher level completed	2.7	0.10 (0.07–0.15)	0.22 (0.14–0.33)	9.5	25.9	14.74 (11.36–19.11)	4.56 (3.37–6.19)
**Employment status**							
Employed	16.1	1 [reference]	1 [reference]	41.7	59.7	1 [reference]	1 [reference]
Unemployed	9.2	1.99 (1.51–2.589)	1.93 (1.46–2.55)	12.0	13.8	0.80 (0.68–0.95)	0.88 (0.72–1.07)
Student	8.2	0.97 (0.32–2.97)	2.98 (0.80–11.12)	2.2	15.3	4.87 (3.58–6.63)	2.06 (1.34–3.1)7
Retired/Disabled	63.0	4.58 (3.80–5.53)	2.44 (1.83–3.26)	35.5	8.6	0.17 (0.15–0.19)	0.73 (0.58–0.92)
Housewife	10.9	3.25 (2.49–4.24)	1.76 (1.30–2.37)	8.6	2.7	0.22 (0.17–0.27)	0.78 (0.60–1.03)
**Size of household (including respondent)**							
1	15.8	1 [reference]	1 [reference]	11.6	6.5	1 [reference]	1 [reference]
2	48.3	0.99 (0.86–1.15)	1.23 (1.05–1.45)	35.6	19.5	0.97 (0.85–1.10)	0.97 (0.82–1.15)
3	21.0	0.58 (0.47–0.70)	1.06 (0.84–1.33)	26.7	32.8	2.18 (1.89–2.51)	1.06 (0.88–1.28)
4	8.9	0.36 (0.28–0.47)	0.86 (0.63–1.18)	17.9	30.1	2.98 (2.54–3.48)	1.05 (0.85–1.30)
>4	5.9	0.52 (0.36–0.77)	1.07 (0.70–1.64)	8.2	11.0	2.38 (1.89–3.00)	0.89 (0.67–1.19)
**Household income per adult**							
Quintile 1	30.1	1 [reference]	1 [reference]	21.9	16.0	1 [reference]	1 [reference]
Quintile 2	28.8	0.94 (0.78–1.13)	0.88 (0.73–1.07)	22.3	15.9	0.98 (0.83–1.15)	1.06 (0.86–1.30)
Quintile 3	20.7	0.72 (0.59–0.87)	0.76 (0.61–0.94)	21.1	19.5	1.27 (1.08– 1.49)	1.31 (1.08–1.60)
Quintile 4	13.4	0.50 (0.40–0.62)	0.58 (0.46–0.73)	19.7	21.9	1.52 (1.30–1.79)	1.40 (1.14–1.71)
Quintile 5	6.9	0.33 (0.26–0.44)	0.49 (0.36–0.66)	15.0	26.8	2.44 (2.07–2.88)	2.09 (1.65–2.66)
**Migration status^c^**							
No	96.1	1 [reference]	1 [reference]	93.0	90.3	1 [reference]	1 [reference]
Yes	3.9	0.54 (0.38–0.77)	1.09 (0.75–1.59)	7.0	9.7	1.42 (1.17–1.73)	0.86 (0.68–1.08)

CI, Confidence interval; OR, Odds ratio.

a Adjusted for sex, age, educational level, degree of urbanization, and presence of chronic diseases.

NUTS II, Nomenclatura de Unidades Territoriais para Fins Estatísticos, nível II (Nomenclature of territorial units for statistics, 2 level); R.A., Região Autónoma (Autonomous Region).

b Based on the share of local population living in urban clusters and in urban centers according to the Commission Directorates-General for Regional and Urban Policy, Agriculture and Rural Development, Eurostat, Joint Research Center and Organization for Economic Co-operation and Development.

c According to country of birth and nationality (No—born in Portugal and having Portuguese nationality, Yes—born in other countries and having Portuguese nationality, or born in Portugal or in other countries and not having Portuguese nationality).

Table 3 presents the association between objective health status and healthcare use and self-perceived health status. Participants with chronic diseases were more likely to report poor/very poor health status (AOR = 5.79, 95%CI: 3.92–8.56), as well as those who had an appointment with a general practitioner (AOR = 1.36, 95%CI: 1.08–1.72), with a specialist doctor (AOR = 1.94, 95%CI: 1.67–2.25), with a psychologist, psychotherapist, or psychiatrist (AOR = 2.17, 95%CI: 1.67–2.79), a hospital admission (AOR = 2.94, 95%CI: 2.41–3.59), or an ambulatory hospital visit (AOR = 2.04, 95%CI: 1.77–2.36) in the last 12 months. Participants who consumed medications with prescription in the previous 2 weeks were more likely to report poor/very poor health status (AOR = 2.33, 95%CI: 1.78–3.05), and similar results were observed for subjects who reported glycaemia (AOR = 1.33, 95%CI: 1.09–1.62) being measured by a health professional in the last 12 months.

**Table 3:** Association between objective health status and healthcare use, and self-perceived health status.

	**Self-perceived health status**						
	**Poor/very poor**			**Fair**	**Good/very good**		
	**Weighted %**	**Crude OR (95%CI)**	**Adjusted^a^ OR (95%CI)**	**Weighted %**	**Weighted %**	**Crude OR (95%CI)**	**Adjusted^a^ OR (95%CI)**
**Chronic disease**							
No	3.4	1 [reference]	1 [reference]	22.6	67.7	1 [reference]	1 [reference]
Yes	96.6	8.33 (5.65–12.26)	5.79 (3.92–8.56)	77.3	32.3	0.14 (0.12–0.16)	0.21 (0.19–0.24)
**Last appointment with general practitioner**							
More than one year ago (includes never)	9.8	1 [reference]	1 [reference]	17.3	33.9	1 [reference]	1 [reference]
In the last year	90.2	1.92 (1.55–2.38)	1.36 (1.08–1.72)	82.7	66.1	0.41 (0.36–0.46)	0.69 (0.59–0.79)
**Last appointment with specialist doctor**							
More than 1 year ago (includes never)	34.6	1 [reference]	1 [reference]	48.4	58.2	1 [reference]	1 [reference]
In the last year	56.4	1.78 (1.55–2.04)	1.94 (1.67–2.)	51.6	41.8	0.67 (0.61–0.74)	0.70 (0.61–0.79)
**Last appointment with a dentist**							
More than 1 year ago (includes never)	69.1	1 [reference]	1 [reference]	55.9	42.5	1 [reference]	1 [reference]
In the last year	30.9	0.57 (0.49–0.66)	0.86 (0.74–1.01)	44.1	57.5	1.72 (1.56–1.90)	1.05 (0.92–1.19)
**Last appointment with a psychologist, psychotherapist, psychiatrist**							
More than 1 year ago (includes never)	89.3	1 [reference]	1 [reference]	93.2	95.8	1 [reference]	1 [reference]
In the last year	10.7	1.63 (1.30–2.05)	2.17 (1.67–2.79)	6.8	4.2	0.60 (0.48–0.75)	051 (0.39–0.67)
**Consumption of medication with prescription in the last 2 weeks**							
No	6.7	1 [reference]	1 [reference]	25.8	66.0	1 [reference]	1 [reference]
Yes	93.3	4.84 (3.74–6.26)	2.33 (1.78–3.05)	74.2	34.0	0.18 (0.16–0.20)	0.47 (0.41– 0.55)
**Consumption of medication without prescription in the last 2 weeks**							
No	79.8	1 [reference]	1 [reference]	77.7	74.1	1 [reference]	1 [reference]
Yes	20.2	0.88 (0.75–1.04)	1.08 (0.90–1.29)	22.2	25.9	1.22 (1.08–1.37)	0.92 (0.80–1.06)
**Hospital admission in the last 12 months**							
No	75.6	1 [reference]	1 [reference]	90.4	95.4	1 [reference]	1 [reference]
Yes	24.4	3.05 (2.54–3.66)	2.94 (2.41–3.59)	9.6	4.6	0.46 (0.37–0.56)	0.54 (0.43–0.68)
**Visits to the hospital for ambulatory health care in the past 12 months**							
No	37.1	1 [reference]	1 [reference]	54.9	68.1	1 [reference]	1 [reference]
Yes	62.9	2.06 (1.80–2.37)	2.04 (1.77–2.36)	45.1	31.9	0.57 (0.52–0.63)	0.60 (0.53–0.68)
**Blood pressure measured by a health professional**							
Never or more than 1 year ago	7.5	1 [reference]	1 [reference]	16.3	31.5	1 [reference]	1 [reference]
In the last year	92.5	2.39 (1.90–3.02)	1.22(1.00–1.50)	83.7	68.5	0.42 (0.37–0.48)	0.65 (0.54–0.78)
**Cholesterol measured by a health professional^b^**							
Never or more than 1 year ago	13.8	1 [reference]	1 [reference]	17.8	28.9	1 [reference]	1 [reference]
In the last year	86.2	1.35 (1.11–1.64)	1.23 (1.00–1.50)	82.2	71.1	0.53 (0.45–0.62)	0.65 (0.54–0.78)
**Glycaemia measured by a health professional^c^**							
Never or more than 1 year	14.3	1 [reference]	1 [reference]	19.4	31.9	1 [reference]	1 [reference]
In the last year	85.7	1.45 (1.20–1.75)	1.33 (1.09–1.62)	80.6	68.1	0.51 (0.44–0.60)	0.65 (0.54–0.77)
**Fecal occult blood test and/or colonoscopy use^d^**							
Never	35.5	1 [reference]	1 [reference]	33.1	43.7	1 [reference]	1 [reference]
Ever (at least once)	64.5	0.90 (0.75–1.09)	0.91 (0.75–1.11)	66.9	56.3	0.64 (0.54–0.75)	0.65 (0.53–0.78)
**Mammography use^e^**							
Never	13.0	1 [reference]	1 [reference]	7.9	8.1	1 [reference]	1 [reference]
Ever (at least once)	87.0	0.57 (0.43–0.76)	0.83 (0.61–1.12)	92.1	91.9	0.97 (0.66–1.42)	0.70 (0.45–1.09)
**Cervical cytology use^f^**							
Never	13.5	1 [reference]	1 [reference]	12.5	13.3	1 [reference]	1 [reference]
Ever (at least once)	86.5	0.91 (0.65–1.28)	1.03 (0.72–1.46)	87.5	86.7	0.93 (0.73–1.17)	0.86 (0.64–1.17)

CI, Confidence interval; OR, Odds ratio.

a Adjusted for sex, age, educational level, degree of urbanization, and presence of chronic diseases.

b Considering only men aged ≥40 years and women aged ≥50 years.

c Considering only subjects aged ≥45 years.

d Considering only subjects aged 50–74 years.

e Considering only women aged 50–69 years.

f Considering only women aged 25–64 years.

Table 4 presents the association between barriers to healthcare services utilization and self-perceived health status. Participants who reported having other healthcare providers besides the national health service were more likely to self-rate their health status as good/very good (AOR = 1.39, 95%CI: 1.18–1.63), as well as individuals who reported having private health insurance (AOR = 1.36, 95%CI: 1.17–1.60). On the contrary, a higher prevalence of poor/very poor health status was reported by participants who reported waiting for a consultation, exam or treatment beyond reasonable (AOR = 1.46, 95%CI: 1.24–1.70) due to distance and/or transportation (AOR = 1.93, 95%CI: 1.34–2.79), or due to financial difficulties (AOR = 1.75, 95%CI: 1.44–2.14). A similar result was observed for those waiting for a dentist consultation, exam, or treatment due to financial difficulties (AOR = 1.57, 95%CI: 1.30–1.90), or in the group of participants not buying medication due to financial difficulties (AOR = 1.79, 95%CI: 1.46–2.19).

**Table 4:** Association between barriers to healthcare services utilization and self-perceived health status.

	**Self-perceived health status**						
	**Poor/very poor**			**Fair**	**Good/very good**		
	**Weighted %**	**Crude OR (95%CI)**	**Adjusted^a^ OR (95%CI)**	**Weighted %**	**Weighted %**	**Crude OR (95%CI)**	**Adjusted^a^ OR (95%CI)**
**Public healthcare provider**							
National health service	88.2	1 [reference]	1 [reference]	84.5	79.8	1 [reference]	1 [reference]
National health service and other subsystems	11.8	0.73 (0.60–0.90)	1.00 (0.80–1.26)	15.5	20.2	1.39 (1.22–1.58)	1.39 (1.18–1.63)
**Private health insurance**							
No	94.4	1 [reference]	1 [reference]	85.2	72.8	1 [reference]	1 [reference]
Yes	5.6	0.34 (0.26–0.45)	0.61 (0.46–0.82)	14.8	27.2	2.15 (1.88–2.45)	1.36 (1.17–1.60)
**Waiting for a consultation, exam or treatment beyond reasonable in the last 12 months**							
No	62.5	1 [reference]	1 [reference]	64.7	62.6	1 [reference]	1 [reference]
Yes	34.1	1.41 (1.22–1.64)	1.46 (1.24–1.70)	25.0	13.7	0.57 (0.59–0.65)	0.56 (0.48–0.66)
No need	3.4	0.34 (0.23–0.48)	0.44 (0.30–0.63)	10.3	23.7	2.39 (2.07–2.76)	1.55 (1.30–1.85)
**Waiting for a consultation, exam or treatment due to distance and/or transportation in the last 12 months**							
No	89.3	1 [reference]	1 [reference]	8.6	75.5	1 [reference]	1 [reference]
Yes	6.4	2.46 (1.79–3.38)	1.93 (1.34–2.79)	2.5	0.50	0.26 (0.17–0.39)	0.34 (0.21–0.54)
No need	4.3	0.36 (0.26–0.49)	0.47 (0.34–0.64)	11.5	24.0	2.37 (2.07–2.72)	1.55 (1.31–1.84)
**Waiting for a medical consultation, exam or treatment due to financial difficulties in the last 12 months**							
No	71.9	1 [reference]	1 [reference]	68.9	59.2	1 [reference]	1 [reference]
Yes	18.4	1.65 (1.38–1.99)	1.75 (1.44–2.14)	10.8	4.7	0.51 (0.42–0.62)	0.60 (0.47–0.75)
No need	10.6	0.50 (0.41–0.62)	0.63 (0.50–0.79)	2.0	36.1	2.07 (1.84–2.33)	1.34 (1.56–1.55)
**Waiting for a dentist consultation, exam or treatment due to financial difficulties in the last 12 months**							
No	27.3	1 [reference]	1 [reference]	37.7	48.0	1 [reference]	1 [reference]
Yes	31.1	1.79 (1.50–2.14)	1.57 (1.30–1.90)	23.9	13.5	0.44 (0.39–0.51)	0.63 (0.53–0.75)
No need	41.6	1.49 (1.27–1.76)	0.98 (0.82–1.17)	38.4	38.5	0.79 (0.71–0.88)	1.12 (0.97–1.29)
**Waiting for a mental health consultation, exam or treatment due to financial difficulties in the last 12 months**							
No	11.2	1 [reference]	1 [reference]	7.0	4.7	1 [reference]	1 [reference]
Yes	7.4	1.24 (0.87–1.77)	1.11 (0.77–1.60)	3.8	0.8	0.33 (0.21–0.53)	0.48 (0.27–0.86)
No need	81.5	0.57 (0.46–0.72)	0.44 (0.35–0.57)	89.2	94.5	1.59 (1.28–1.97)	1.78 (1.36–2.32)
**Not buying medication due to financial difficulties in the last 12 months**							
No	75.9	1 [reference]	1 [reference]	74.8	58.9	1 [reference]	1 [reference]
Yes	17.5	1.85 (1.52–2.24)	1.79 (1.46–2.19)	9.3	3.7	0.51 (0.40–0.63)	0.69 (0.53–0.89)
No need	6.6	0.41 (0.32–0.53)	0.62 (0.47–0.82)	15.8	37.5	3.00 (2.65–3.41)	1.60 (1.37–1.87)

CI, Confidence interval; OR, Odds ratio.

a Adjusted for sex, age, educational level, degree of urbanization, and presence of chronic diseases.

Table 5 shows the association between activity limitation and lifestyle factors, and self-perceived health status. A marked higher prevalence of poor/very poor health status was reported by participants with severe activity limitation (AOR = 21.29, 95%CI: 16.46–27.55) or who have limited but not severe activity limitation (AOR = 4.91, 95%CI: 4.00–6.06), compared with those without activity limitation. Participants who reported absence to work due to individual health problems were also more likely to self-rate their health status as poor/very poor (AOR = 2.76, 95%CI: 1.96–3.90). There was a trend toward worse health status with increasing intensity of pain felt by participants in the previous 4 weeks. Moreover, a similar trend was observed according to interference from pain in the usual tasks. A lack of consistent associations was observed between lifestyles and self-perceived health status, except for current tobacco smoking and intake of 5 portions or more per day of fruits and vegetables, which were significantly associated with poor/very poor health status (AOR = 1.44, 95%CI: 1.13–1.86 and AOR = 0.76, 95%CI: 0.62–0.92, respectively).

**Table 5:** Association between activity limitation and lifestyle factors, and self-perceived health status.

	**Self-perceived health status**						
	**Poor/very poor**			**Fair**	**Good/very good**		
	**Weighted %**	**Crude OR (95%CI)**	**Adjusted^a^ OR (95%CI)**	**Weighted %**	**Weighted %**	**Crude OR (95%CI)**	**Adjusted^a^ OR (95%CI)**
**General activity limitation**							
Not limited	12.4	1 [reference]	1 [reference]	59.6	89.1	1 [reference]	1 [reference]
Limited but not severely	48.3	6.76 (5.53–8.25)	4.91 (4.00–6.04)	34.3	9.3	0.18 (0.16–0.21)	0.35 (0.30–0.41)
Severely limited	39.3	30.75 (24.04–39.33)	21.29 (16.46–27.55)	6.1	1.6	0.17 (0.12–0.23)	0.31 (0.21–0.44)
**Absence to work for individual health problems**							
No	40.8	1 [reference]	1 [reference]	65.7	80.2	1 [reference]	1 [reference]
Yes	59.2	2.77 (1.97–3.91)	2.76 (1.96–3.90)	34.3	19.8	0.47 (0.40–0.56)	0.52 (0.43–0.63)
**Intensity of pain felt in the previous 4 weeks**							
No	13.2	1 [reference]	1 [reference]	33.2	64.3	1 [reference]	1 [reference]
Very slight/slight	15.4	1.33 (1.05–1.69)	1.11 (0.86–1.42)	29.0	22.1	0.39 (0.35–0.45)	0.49 (0.42–0.57)
Moderate	20.5	2.47 (1.97–3.11)	1.78 (1.38–2.27)	20.7	8.3	0.21 (0.18–0.24)	0.36 (0.30–0.44)
Intense/very intense	5.3	7.43 (5.97–9.24)	5.59 (4.42–7.06)	17.1	5.3	0.16 (0.13–0.19)	0.29 (0.23–0.36)
**Interference from pain in the usual tasks**							
Nothing	18.8	1 [reference]	1 [reference]	52.4	84.0	1 [reference]	1 [reference]
A little/moderately	40.1	2.91 (2.43–3.49)	2.34 (1.93–2.82)	38.5	14.2	0.23 (0.20–0.026)	0.35 (0.30–0.40)
Very/in an extreme way	41.2	12.65 (10.28–15.57)	9.54 (7.65–11.89)	9.1	1.8	0.12 (0.09–0.16)	0.24 (0.18–0.32)
**Body mass index by categories (kg/m^2^)**							
18.5–24.9	31.6	1 [reference]	1 [reference]	35.3	55.5	1 [reference]	1 [reference]
<18.5	2.2	2.01 (1.16–3.48)	2.93 (1.60–5.37)	1.2	3.2	1.69 (1.11–2.57)	0.93 (0.58–1.50)
25.0–29.9	39.9	1.06 (0.91–1.25)	0.81 (0.68–0.96)	41.8	31.4	0.48 (0.43–0.53)	0.79 (0.69–0.91)
≥30.0	26.3	1.35 (1.13–1.62)	1.05 (0.86–1.29)	21.7	9.9	0.29 (0.25–0.34)	0.57 (0.47–0.68)
**Condition on tobacco consumption**							
Never	68.2	1 [reference]	1 [reference]	59.6	54.3	1 [reference]	1 [reference]
Former	19.9	0.75 (0.63–0.89)	1.17 (0.95–1.45)	23.2	21.5	1.02 (0.90–1.15)	0.99 (0.85–1.16)
Current	11.8	0.60 (0.48–0.75)	1.44 (1.13–1.86)	17.2	24.2	1.55 (1.36–1.76)	0.87 (0.74–1.03)
**Drinking status**							
Never	30.5	1 [reference]	1 [reference]	21.5	17.7	1 [reference]	1 [reference]
Former	20.4	1.42 (1.16–1.74)	1.23 (0.99–1.52)	10.2	5.5	0.65 (0.53–0.80)	0.95 (0.72–1.24)
Current	49.1	0.51 (0.44–0.59)	0.63 (0.52–0.75)	68.3	76.8	1.37 (1.21–1.55)	1.19 (1.01–1.41)
**Fruits and vegetables (portions per day)**							
<5	85.4	1 [reference]	1 [reference]	80.9	81.7	1 [reference]	1 [reference]
≥5	14.6	0.73 (0.61–0.87)	0.76 (0.62–0.92)	19.1	18.3	0.95 (0.83–1.08)	1.17 (1.00–1.37)

CI, Confidence interval; OR, Odds ratio.

a Adjusted for sex, age, educational level, degree of urbanization, and presence of chronic diseases.

Table 6 presents the association between mental health, social support and satisfaction with life, and self-perceived health status. A higher prevalence of poor/very poor health status was reported by participants who had depressed mood (AOR = 3.03, 95%CI: 2.64–3.62), sleep disorders (AOR = 2.10, 95%CI: 1.80–2.44), fatigue (AOR = 3.61, 95%CI: 3.03–4.29), appetite change (AOR = 2.75, 95%CI: 2.33–3.24), feeling of uselessness or guilt (AOR = 3.09, 95%CI: 2.65–3.60), difficulty concentrating (AOR = 2.83, 95%CI: 2.42–3.31) in the 2 weeks preceding the survey. There was a trend toward worse health status with a decreasing number of persons close to the participant whom they could seek in the event of a serious personal problem. Participants who reported a low level of concern or interest of other people in relation to them had a higher risk of reporting poor/very poor health status (AOR = 1.46, 95%CI: 1.04–2.05), in comparison to participants who reported higher levels of interest. Participants who expressed having difficulty getting help from neighbors in case of need were more likely to report poor/very poor health status (AOR = 1.32, 95%CI: 1.11–1.59), in comparison to individuals who reported easier access to their neighbors. An increase in the likelihood of reporting poor/very poor self-perceived health was observed among participants who were in disagreement or had a neutral opinion about self-appreciation of proximity to their ideals of life (AOR = 2.64, 95%CI: 2.24–3.10 and AOR = 1.89, 95%CI: 1.53–2.33, respectively), satisfaction with living conditions (AOR = 2.81, 95%CI: 2.40–3.29 and AOR = 1.63, 95%CI: 1.28–2.07, respectively), life satisfaction (AOR = 3.37, 95%CI: 2.86–3.97 and AOR = 1.69, 95%CI: 1.33–2.16, respectively), obtaining the most important in life (AOR = 2.05, 95%CI: 1.73–2.43 and AOR = 1.67, 95%CI: 1.29–2.17, respectively), and self-appreciation with the life-path satisfaction (AOR = 1.73, 95%CI: 1.48–2.01 and AOR = 1.66, 95%CI: 1.28–2.15, respectively), when compared with participants who were in agreement.

**Table 6:** Association between mental health, social support and satisfaction with life, and self-perceived health status.

	**Self-perceived health status**						
	**Poor/very poor**			**Fair**	**Good/very good**		
	**Weighted %**	**Crude OR (95%CI)**	**Adjusted^a^ OR (95%CI)**	**Weighted %**	**Weighted %**	**Crude OR (95%CI)**	**Adjusted^a^ OR (95%CI)**
**Depressed mood frequency in the last 2 weeks**							
Never	32.0	1 [reference]	1 [reference]	60.7	83.8	1 [reference]	1 [reference]
Ever	68.0	3.28 (2.85–3.79)	3.09 (2.64–3.62)	39.3	16.2	0.30 (0.27–0.34)	0.39 (0.33–0.44)
**Frequency of sleep disorders in the last 2 weeks**							
Never	30.6	1 [reference]	1 [reference]	51.1	70.3	1 [reference]	1 [reference]
Ever	69.4	2.36 (2.05–2.72)	2.10 (1.80–2.44)	48.9	29.7	0.42 (0.38–0.47)	0.52 (0.46–0.60)
**Frequency of fatigue in the last 2 weeks**							
Never	18.1	1 [reference]	1 [reference]	45.0	66.0	1 [reference]	1 [reference]
Ever	81.9	3.72 (3.16–4.38)	3.61 (3.03–4.29)	55.0	34.0	0.42 (0.38–0.47)	0.42 (0.37–0.47)
**Frequency of appetite change in the last 2 weeks**							
Never	61.3	1 [reference]	1 [reference]	89.8	89.7	1 [reference]	1 [reference]
Ever	38.7	2.67 (2.30–3.10)	2.75 (2.33–3.24)	19.2	10.3	0.49 (0.42–0.56)	0.43 (0.36–0.52)
**Frequency of feeling of uselessness or guilt in the last 2 weeks**							
Never	47.6	1 [reference]	1 [reference]	75.0	90.9	1 [reference]	1 [reference]
Ever	52.4	3.30 (2.86–3.79)	3.09 (2.65–3.60)	25.0	9.1	0.30 (0.26–0.35)	0.39 (0.33–0.47)
**Frequency of difficulty concentrating in the last 2 weeks**							
Never	57.2	1 [reference]	1 [reference]	80.3	91.4	1 [reference]	1 [reference]
Ever	42.8	3.06 (2.64–3.54)	2.83 (2.42–3.31)	19.7	8.6	0.38 (0.33–0.44)	0.44 (0.37–0.53)
**Number of persons close to the participant whom they could seek in the event of a serious personal problem**							
6 or more	17.4	1 [reference]	1 [reference]	21.3	27.2	1 [reference]	1 [reference]
3 to 5	33.4	1.06 (0.87–1.30)	1.06 (0.86–1.31)	38.6	44.5	0.91 (0.79–1.04)	0.88 (0.74–1.03)
1 to 2	44.4	1.46 (1.20–1.78)	1.34 (1.09–1.64)	37.4	27.0	0.57 (0.49–0.65)	0.75 (0.63–0.88)
No	4.8	2.20 (1.52–3.16)	1.98 (1.31–2.97)	2.7	1.3	0.38 (0.26–0.53)	0.68 (0.44–1.06)
**Level of concern or interest of other people in relation to the participant**							
Some concern and interest/a lot of concern and interest	88.6	1 [reference]	1 [reference]	88.3	91.0	1 [reference]	1 [reference]
Cannot evaluate	6.3	0.75 (0.57–0.97)	0.77 (0.58–1.02)	8.4	7.4	0.86 (0.71–1.03)	0.92 (0.74–1.16)
No concern and interest/little concern and interest	5.1	1.53 (1.11–2.11)	1.46 (1.04–2.05)	3.3	1.6	0.46 (0.34–0.63)	0.72 (0.49–1.04)
**Degree of perception of getting help from neighbors in case of need**							
Very easy/easy	51.2	1 [reference]	1 [reference]	49.3	46.6	1 [reference]	1 [reference]
Possible	22.1	0.74 (0.63–0.87)	0.92 (0.77–1.10)	28.8	33.4	1.23 (1.09–1.38)	0.88 (0.77–1.02)
Difficult/very difficult	26.7	1.17 (0.99–1.39)	1.32 (1.11–1.59)	21.9	20.1	0.97 (0.85–1.11)	0.85 (0.72–1.01)
**Self–appreciation of proximity to the participant's ideals of life**							
More or less in agreement/in agreement/totally in agreement	41.9	1 [reference]	1 [reference]	64.1	76.7	1 [reference]	1 [reference]
Neither in agreement nor in disagreement	15.6	2.05 (1.67–2.51)	1.89 (1.53–2.33)	11.7	7.5	0.54 (0.45–0.64)	0.59 (0.49–0.72)
Totally in disagreement/in disagreement/more or less in disagreement	42.5	2.69 (2.32–3.12)	2.64 (2.24–3.10)	24.1	15.7	0.54 (0.48–0.62)	0.65 (0.56–0.76)
**Self–appreciation of the participant's satisfaction with living conditions**							
More or less in agreement/in agreement/totally in agreement	36.4	1 [reference]	1 [reference]	56.1	72.4	1 [reference]	1 [reference]
Neither in agreement nor in disagreement	11.3	1.57 (1.25–1.98)	1.63 (1.28–2.07)	11.1	8.9	0.62 (0.53–0.74)	0.61 (0.49–0.76) 0.47 (0.41–0.55)
Totally in disagreement/in disagreement/more or less in disagreement	52.4	2.45 (2.13–2.83)	2.81 (2.40–3.29)	32.9	18.7	0.44 (0.39–0.50)	
**Self–appreciation of the participant's life satisfaction**							
More or less in agreement/in agreement/totally in agreement	47.8	1 [reference]	1 [reference]	71.4	83.5	1 [reference]	1 [reference] 0.61 (0.48–0.77)
Neither in agreement nor in disagreement	9.6	1.71 (1.34–2.16)	1.69 (1.33–2.16)	8.7	5.8	0.57 (0.47–0.69)	
Totally in disagreement/in disagreement/more or less in disagreement	42.3	3.17 (2.73–3.68)	3.37 (2.86–3.97)	19.9	10.7	0.46 (0.40–0.53)	0.54 (0.45–0.64)
**Self-appreciation of obtaining the most important in life**							
More or less in agreement/in agreement/totally in agreement	59.6	1 [reference]	1 [reference]	72.5	82.8	1 [reference]	1 [reference]
Neither in agreement nor in disagreement	11.0	1.62 (1.27–2.05)	1.67 (1.29–2.17)	8.3	5.1	0.54 (0.44–0.66)	0.48 (0.37–0.62)
Totally in disagreement/in disagreement/more or less in disagreement	29.4	1.85 (1.59–2.17)	2.05 (173–2.43)	19.3	12.1	0.55 (0.48–0.64)	0.51 (0.43–0.61)
**Self-appreciation with the life-path satisfaction**							
More or less in agreement/in agreement/totally in agreement	40.9	1 [reference]	1 [reference]	52.3	63.9	1 [reference]	1 [reference]
Neither in agreement nor in disagreement	9.6	1.85 (1.43–2.38)	1.66 (1.28–2.15)	6.6	7.3	0.90 (0.74–1.10)	0.99 (0.77–1.27)
Totally in disagreement/in disagreement/more or less in disagreement	49.5	1.53 (1.33–1.77)	1.73 (1.48–2.01)	41.1	28.8	0.57 (0.52–0.64)	0.62 (0.55–0.71)

CI, Confidence interval; OR, Odds ratio.

a Adjusted for sex, age, educational level, degree of urbanization, and presence of chronic diseases.

## Discussion

This paper provides broad guidance on factors that can affect self-perceived health status in Portugal by using representative data collected at the National Health Survey of 2014. Our results show variability in the patterns of self-perceived health, which translates into marked socioeconomic, healthcare use, lifestyle, mental and physical inequalities among participants with poorer self-perceived health.

Almost half of the participants tended to rate their health as good or very good, and nearly one-third rated their health as fair, while about one-sixth rated their health as poor or very poor. In 2016, a summary of self-perceived health status showed that the overall perceived health among the European Union population was 67.5 % as very good or good, 23.7 % as fair, and 8.8 % as poor or very poor (42). This means that subjects living in Portugal tend to report less good/very good health status and more poor/very poor health when compared to Europe, which is also in line with another study that showed Portugal having one of the greatest percentages of poor self-perceived health in Europe (6, 14).

The gender gap in self-perceived health status, which translates into women tending to rate their health worse than men, is in line with self-perceived health status in the entire EU, in which women exhibit higher rates of poor/very poor health when compared to men (43). The largest health status gender gaps were recorded in Portugal, Romania, Latvia, and Lithuania (43), and these findings are also in line with previous studies in several countries, including Portugal (6, 44). One reading of this would be men are less likely to exhibit suffering or pain when compared to women (45). Moreover, males have a considerably higher risk of fatal injury and sudden death than females rather than disability in almost all age groups in the EU (46). The years of life lost among men in the EU before the age of 65 are twice as women (47), while in Portugal, mortality among men aged 15–34 years is more than three times higher than mortality among women in the same age category (47).

The poor self-perceived health among older age categories in our study clearly demonstrates the importance of providing and maintaining healthy aging in Portugal. This result is in line with previous studies that showed that both health status and self-perceived health deteriorates in the elderly (48, 49). The high levels of poor perceived health among elderly can be explained by how age can influence functional ability and welfare (50, 51) and by the dynamic measure of self-perceived health, which is accounting for assessment of the path/course of future health and not merely the current health status (52).

Our study identified socioeconomic inequalities as important factors associated with poor self-perceived health in Portugal. This finding is in line with several studies that documented the positive correlation between socioeconomic and health status (53, 54). Moreover, it is widely known that socioeconomic position plays a substantial role in shaping health inequalities, particularly in Portugal (55). These results are in accordance with previous studies that documented a strong positive association between education (56), employment (57), income (58), marital status (59), household composition (59), and self-perceived health. Since previous studies concluded that individual factors that predominantly consisted of socio-economic indicators are of paramount importance, as they may account for 90% of differences in health status (56).

Our study also assessed the evidence concerning the place of living as an indicator of self-perceived health inequalities. This finding is not surprising given the well-known geographical inequalities in terms of the distribution of health services, allocation of medical resources, and variations in the socioeconomic status among different areas in Portugal. For example, specific regions of the country, particularly the coastal regions, exhibit better economic growth rates and health outcomes (55). On the contrary, less developed areas exhibit lower accessibility to health services and healthcare utilization (55). Finally, medical and human resources are primarily concentrated in large main cities, namely, Lisbon, Porto, and Coimbra (55).

All factors that measure illness or indicate recent healthcare use or disability were linked to a high probability of reporting poor health status. Other studies reported similar findings in which chronic illness and comorbidities significantly increased the reporting of poor self-perceived health (60, 61). This finding can be explained by the effect of morbidities, which can go beyond the medical and biological to affect daily activities (51, 62). Morbidity by itself may disturb functional, coping, and wellbeing factors, and accordingly, change the way patients may perceive their health which supports the evidence of considering health perception as a multidimensional construct that can be affected by several factors beyond physical wellbeing (51).

Barriers to access healthcare services were considerably associated with reporting poor self-perceived health. This finding is not surprising since higher access to health care was always linked to better health outcomes (63). Transportation (64), financial constraints (65, 66), and improper healthcare (67, 68) are usually defined as barriers to access healthcare services. These barriers may result in delayed care or medical treatment resulting in disease exacerbations and poorer health outcomes, especially when it comes to chronic disease that requires regular follow-up or sometimes adjustments for treatment protocols with regard to offering appropriate care (64, 66).

As for lifestyle indicators, and despite the well-documented consequences of these behaviors on health and self-perceived health (4, 23), our results showed a lack of consistent association between alcohol drinking, obesity, and poor self-perceived health. However, this lack of consistent association could be expected, given the high prevalence of obesity (69) and alcohol consumption (70) in Portugal. However, and as expected, tobacco smoking was strongly associated with reporting poor health. The devastating effect of tobacco smoking on both health and self-perceived health is well-documented in previous studies (23). On the contrary, a high intake of fruits and vegetables was less likely to be associated with reporting poor/very poor self-perceived health, which agrees with previous studies that linked the high frequency of eating fruits and vegetables to good health (71).

The association between mental illness and poor self-perceived health generally agrees with those obtained in previous studies (72, 73). The fundamental link between mental health, physical health, and functional disability that has two pathways from one to the other may provide a conceptual framework. On the one hand, mental illness by itself can represent a risk factor for chronic illness (37, 38). On the other hand, individuals with chronic conditions have higher rates of mental illness (74, 75). In others, chronic medical illness may affect a patient's functional ability and independence and alter the way patients live. However, another study found that individuals' self-perceived health kept strongly associated with depression despite controlling for morbidities and functional disability (76).

Our study also identified socially isolated individuals as more susceptible to reporting poor health status. High degrees of social capital have a protective role with regard to perceptions of health (17), especially in case of constraints due to chronic illness or acute incidences (77). Moreover, poor health outcomes linked to social deprivation are adequately assessed in previous studies, in which socially deprived individuals tend to exhibit higher death rates (78, 79) and illness (79, 80). On how social isolation can affect health, ample evidence suggests that socially deprived have less access to supportive factors, such as information and emotional support (79), which may affect their ability to active cope with stresses (43, 79), control over life, and self-esteem (79, 81).

This study emphasizes the importance of implying a wide range of factors that can impact self-perceived health status. Considering these factors in clinical practices and policy interventions may contribute to better health outcomes given their ability to predict self-perceived health status. For example, policies should be directed toward addressing the effect of socio-economic disparities on health given their significant impact. Findings from our study demand further analysis of disparities by gender, education, income, and region that can be linked to poor health outcomes given the well-documented socioeconomic inequalities in Portugal. Moreover, results from our study should urge health policymakers to consider how to care for the growing elderly population in Portugal. One possible solution that may have policy implications among the elderly is chronic disease self-management programs. This program implies self-management education for patients with chronic diseases and aims to increase their capability to maintain good health. The program has proven to assist patients in improving their health conditions and behaviors (82).

The significant association between the lack of access to healthcare and poor health status in our study requires further investigation of barriers experienced by Portuguese. Further research is mandatory to analyze the nature of these barriers in order to establish a framework for solving these issues. In addition, policymakers must adopt strategies to mitigate the effects of these barriers on health outcomes. Also, we believe that policies that consider community health needs assessment to target these barriers can contribute to the solutions.

The lack of life satisfaction has been identified as an important factor for poor health perception in our study. This finding is supported by a growing body of evidence that self-perceived health is strongly associated with life satisfaction (19), with satisfaction with life being the main driver of poor perceived health, outranking other factors such as somatic and psychiatric conditions (16). It is important to mention that questions regarding satisfaction with life were first introduced in the Portuguese National Health Survey of 2014, hence, this study provides a relatively recent glimpse on the association between satisfaction with life and self-perceived health.

Since mental health is instrumental in determining self-perceived health, comprehensive plans addressing mental health are mandatory. Portugal is recording one of the highest prevalence of mental illness problems compared to other European countries (83, 84). Despite this fact, most patients with mental illness do not have access to mental health services (85). In addition, mental health services in Portugal have substantial insufficiencies regarding equity and quality of care (85).

The main limitation of this study is the cross-sectional character of the survey, in which a causal relation between self-perceived health status and independent factors cannot be established due to the lack of the time sequence. A reverse causality bias could occur as some of the factors associated with self-perceived health status may be consequences instead of causes. However, the main strength of this study is the use of a large nationally representative sample that has been collected through household sampling. Moreover, this study was able to identify a wide range of self-perceived health risk factors with implications for policies aiming to improve overall health.

## Conclusion

This study adds to previous research on how self-perceived health status can be affected by several factors that include socioeconomic status, chronic illness, mental health, access to health services, satisfaction with life, and social support. This study has implications for policies aiming to reduce health inequalities among subjects living in Portugal, illustrating variations in and determining obstacles toward maintaining a better health perception at a national level. Further distinctive work is required to study causal determinants for health perception inequalities and implement and adopt new strategies for better planning of health care.

## Data availability statement

The original contributions presented in the study are included in the article/supplementary files, further inquiries can be directed to the corresponding author.

## Ethics statement

The project was approved by the Portuguese National Authority of Data Protection for the creation of an individual database and by the Ethics Committees of the participant entities. All subjects inquired signed informed consent. Our specific study was approved by scientific committee of National Institute of Statistics/Portuguese Foundation for Science and Technology/Direção-Geral de Estatísticas da Educação e Ciência (process nr. 711). The patients/participants provided their written informed consent to participate in this study.

## Author contributions

AS and BP performed all the analyses and drafted the manuscript. BP and MM discussed all the analyses and interpretations, and revised the manuscript. All authors read and approved the final version of the manuscript.

## Funding

This study was funded by the Foundation for Science and Technology—FCT (Portuguese Ministry of Science, Technology and Higher Education) under the Department of Global Health and Tropical Medicine (GHTM)—The Portuguese Institute of Hygiene and Tropical Medicine (IHMT)—NOVA University of Lisbon (UNL); the Ph.D. Grants PD/BD/128066/2016 (AS) co-funded by FCT, the IHMT, FCT, and the POCH/FSE Program, FEDER through the Operational Programme Competitiveness and Internationalization and national funding from the Foundation for Science and Technology—FCT (Portuguese Ministry of Science, Technology and Higher Education) under the *Unidade de Investigação em Epidemiologia—Instituto de Saúde Pública da Universidade do Porto* (EPIUnit) (POCI- 01-0145-FEDER-006862; Ref. UID/DTP/04750/2013).

## Conflict of interest

The authors declare that the research was conducted in the absence of any commercial or financial relationships that could be construed as a potential conflict of interest.

## Publisher's note

All claims expressed in this article are solely those of the authors and do not necessarily represent those of their affiliated organizations, or those of the publisher, the editors and the reviewers. Any product that may be evaluated in this article, or claim that may be made by its manufacturer, is not guaranteed or endorsed by the publisher.

## Abbreviations

BMI: Body Mass Index
EU: European Union
INE: Instituto Nacional de Estatística
ISCED: International Standard Classification of Education
NUTS II: Nomenclatura de Unidades Territoriais para Fins Estatísticos, nível II (Nomenclature of territorial units for statistics, 2 level)
PSU: Primary Sampling Units
R.A.: Região Autónoma (Autonomous Region)
UNESCO: United Nations Educational, Scientific and Cultural Organization
WHO: World Health Organization.

## References

[1] Krause NM Jay GM. What do global self-rated health items measure? Med Care. 1994:930–42. 10.1097/00005650-199409000-00004 8090045

[2] Jylhä M. What is self-rated health and why does it predict mortality? Towards a unified conceptual model. Soc Sci Med. (2009) 69:307–16. 10.1016/j.socscimed.2009.05.013 19520474

[3] Denche-Zamorano Á Mendoza-Muñoz M Carlos-Vivas J Muñoz-Bermejo L Rojo-Ramos J Pastor-Cisneros R. A cross-sectional study on self-perceived health and physical activity level in the Spanish population. Int J Environ Res Public Health. (2022) 19:5656. 10.3390/ijerph19095656 35565051 PMC9104406

[4] Ferri-García R Rueda MdM Cabrera-León A. Self-perceived health, life satisfaction and related factors among healthcare professionals and the general population: analysis of an online survey, with propensity score adjustment. Mathematics. (2021) 9:791. 10.3390/math9070791

[5] Gorabi AM Heshmat R Farid M Motamed-Gorji N Motlagh ME Zavareh NH-T. Economic inequality in life satisfaction and self-perceived health in Iranian children and adolescents: the CASPIAN IV study. Int J Prev Med. (2019) 10:70. 10.4103/ijpvm.IJPVM_508_17 31198505 PMC6547786

[6] Borrell C Espelt A Rodríguez-Sanz M Burström B Muntaner C Pasarín MI. Analyzing differences in the magnitude of socioeconomic inequalities in self-perceived health by countries of different political tradition in Europe. Int J Health Serv. (2009) 39:321–41. 10.2190/HS.39.2.f 19492628

[7] Nogueira H Santana P Santos R. Linking perceptions of health to neighbourhood environment in the Lisbon Metropolitan Area, Portugal. WIT Trans Ecol Environ. (2006) 93: 723–31. 10.2495/SC060691

[8] Figueiredo JPd Cardoso SM. Perceived health in the Portuguese population aged? 35. Revista Saude Publica. (2014) 48:406–27. 10.1590/S0034-8910.2014048005242 25119936 PMC4203081

[9] Fonseca H Gaspar de Matos M. Perception of overweight and obesity among Portuguese adolescents: an overview of associated factors. Eur J Public Health. (2005) 15:323–8. 10.1093/eurpub/cki071 15905184

[10] Mota J Santos RM Silva P Aires L Martins C Vale S. Associations between self-rated health with cardiorespiratory fitness and obesity status among adolescent girls. J Phys Activity Health. (2012) 9:378–81. 10.1123/jpah.9.3.378 22454438

[11] Silva PAd. Individual and social determinants of self-rated health and well-being in the elderly population of Portugal. Cadernos Saude Publica. (2014) 30:2387–400. 10.1590/0102-311X00173813 25493992

[12] Prazeres F Santiago L. Relationship between health-related quality of life, perceived family support and unmet health needs in adult patients with multimorbidity attending primary care in Portugal: a multicentre cross-sectional study. Health Qual Life Outcomes. (2016) 14:156. 10.1186/s12955-016-0559-7 27835995 PMC5106778

[13] Pacheco-Figueiredo L Lunet N. Health status, use of healthcare, and socio-economic implications of cancer survivorship in Portugal: results from the Fourth National Health Survey. J Cancer Survivorship. (2014) 8:611–7. 10.1007/s11764-014-0370-6 24903019

[14] Carvalho ATd Malta DC Barros M Oliveira P Mendonça D Barros H. Inequalities in self-rated health: an analysis of the Brazilian and Portuguese populations. Cad Saude Publica. (2015) 31:2449–61. 10.1590/0102-311X00108814 26840823

[15] Ormel J Kempen GI Deeg DJ Brilman EI van Sonderen E Relyveld J. Functioning, well-being, and health perception in late middle-aged and older people: comparing the effects of depressive symptoms and chronic medical conditions. J Am Geriatr Soc. (1998) 46:39–48. 10.1111/j.1532-5415.1998.tb01011.x 9434664

[16] Al-Windi A. The relations between symptoms, somatic and psychiatric conditions, life satisfaction and perceived health. A primary care based study. Health Qual Life Outcomes. (2005) 3:28. 10.1186/1477-7525-3-28 15857513 PMC1131915

[17] Shields M. Community belonging and self-perceived health. Health Rep. (2008) 19:51–60. 18642519

[18] Kawachi I Kennedy BP Glass R. Social capital and self-rated health: a contextual analysis. Am J Public Health. (1999) 89:1187–93. 10.2105/AJPH.89.8.1187 10432904 PMC1508687

[19] An J-Y An K O'Connor L Wexler S. Life satisfaction, self-esteem, and perceived health status among elder Korean women: focus on living arrangements. J Transcult Nurs. (2008) 19:151–60. 10.1177/1043659607313070 18362207

[20] Paul P Hakobyan M Valtonen H. The association between self-perceived health status and satisfaction with healthcare services: evidence from Armenia. BMC Health Serv Res. (2016) 16:67. 10.1186/s12913-016-1309-6 26892950 PMC4759944

[21] Cott CA Gignac MA Badley EM. Determinants of self rated health for Canadians with chronic disease and disability. J Epidemiol Community Health. (1999) 53:731–6. 10.1136/jech.53.11.731 10656104 PMC1756802

[22] Idler EL Kasl SV. Self-ratings of health: do they also predict change in functional ability? J Gerontol Ser B Psychol Sci Soc Sci. (1995) 50:S344–53. 10.1093/geronb/50B.6.S344 7583813

[23] Johnson PB Richter L. The relationship between smoking, drinking, and adolescents' self-perceived health and frequency of hospitalization: analyses from the 1997 National Household Survey on Drug Abuse. J Adolesc Health. (2002) 30:175–83. 10.1016/S1054-139X(01)00317-2 11869924

[24] Okosun IS Choi S Matamoros T Dever GA. Obesity is associated with reduced self-rated general health status: evidence from a representative sample of white, black, and Hispanic Americans. Prev Med. (2001) 32:429–36. 10.1006/pmed.2001.0840 11330993

[25] Instituto Nacional de Estatatistica. Documento metodologico do inquerito nacional de saude 2014 [methodological document of the national health survey 2014]. Lisbon: INE (2014).

[26] Instituto Nacional de Estatistica. Inquerito nacional de saude 2014 [national health survey 2014]. Lisbon: INE (2016).

[27] Idler EL Benyamini Y. Self-rated health and mortality: a review of twenty-seven community studies. J Health Soc Behav. (1997). 38:21–37. 10.2307/2955359 9097506

[28] Eurostat. Glossary:Minimum European Health Module (MEHM). Available online at: https://ec.europa.eu/eurostat/statistics-explained/index.php?title=Glossary:Minimum_European_Health_Module_(MEHM)#:~:text=The%20Minimum%20European%20Health%20Module,is%20your%20health%20in%20general%3F (accessed July 20, 2022).

[29] European Commission Eurostat NUTS - Nomenclature of territorial units for statistics. Available online at: https://ec.europa.eu/eurostat/web/nuts/background (accessed April 14, 2020).

[30] Eurostat. Degree of urbanisation. Available online at: https://ec.europa.eu/eurostat/web/degree-of-urbanisation/background (accessed March 14, 2020).

[31] Eurostat. Income quintile group. Available online at: https://ec.europa.eu/eurostat/statistics-explained/index.php/Glossary:Income_quintile_group (accessed March 14, 2020).

[32] Eurostat. Equivalised disposable income. Available online at: https://ec.europa.eu/eurostat/statistics-explained/index.php?title=Glossary:Equivalised_disposable_income (accessed March 14, 2020).

[33] United Nations Educational S, Organization C. International Standard Classification of Education ISCED 2011. Montreal: UNESCO Institute for Statistics Montreal (2012).

[34] Cutler DM Lleras-Muney A. Education and health: evaluating theories and evidence. National bureau of economic research (2006). Report No.: 0898–2937. 10.3386/w12352

[35] Baker DW Sudano JJ Albert JM Borawski EA Dor A. Lack of health insurance and decline in overall health in late middle age. N Engl J Med. (2001) 345:1106–12. 10.1056/NEJMsa002887 11596591

[36] Padrão P Lunet N Santos AC Barros H. Smoking, alcohol, and dietary choices: evidence from the Portuguese National Health Survey. BMC Public Health. (2007) 7:138. 10.1186/1471-2458-7-138 17608935 PMC1939992

[37] Scherrer JF Garfield LD Chrusciel T Hauptman PJ Carney RM Freedland KE. Increased risk of myocardial infarction in depressed patients with type 2 diabetes. Diabetes Care. (2011) 34:1729–34. 10.2337/dc11-0031 21680721 PMC3142034

[38] Correll CU Solmi M Veronese N Bortolato B Rosson S Santonastaso P. Prevalence, incidence and mortality from cardiovascular disease in patients with pooled and specific severe mental illness: a large-scale meta-analysis of 3,211,768 patients and 113,383,368 controls. World Psychiatry. (2017) 16:163–80. 10.1002/wps.20420 28498599 PMC5428179

[39] Uchino BN. Social support and health: a review of physiological processes potentially underlying links to disease outcomes. J Behav Med. (2006) 29:377–87. 10.1007/s10865-006-9056-5 16758315

[40] Diener E. Assessing subjective well-being: progress and opportunities. Soc Indicat Res. (1994) 31:103–57. 10.1007/BF01207052

[41] Koivumaa-Honkanen H Honkanen R Viinamäki H Heikkilä K Kaprio J Koskenvuo M. Self-reported life satisfaction and 20-year mortality in healthy Finnish adults. Am J Epidemiol. (2000) 152:983–91. 10.1093/aje/152.10.983 11092440

[42] Self-perceived health statistics: Eurostat. Available online at: https://ec.europa.eu/eurostat/statistics-explained/index.php/Self-perceived_health_statistics (accessed July 22, 2022).

[43] Waite LJ Hughes ME. At risk on the cusp of old age: living arrangements and functional status among black, white and Hispanic adults. J Gerontol Ser B Psychol Sci Soc Sci. (1999) 54:S136–44. 10.1093/geronb/54B.3.S136 10363044 PMC1201047

[44] Gilmore AB McKee M Rose R. Determinants of and inequalities in self-perceived health in Ukraine. Soc Sci Med. (2002) 55:2177–88. 10.1016/S0277-9536(01)00361-6 12409131

[45] Robertson S. Understanding Men and Health: Masculinities, Identity and Well-being. Berkshire: McGraw-Hill Education (UK) (2007).

[46] Prevention EAfI Promotion S. Injuries in the European Union. Summary of injury statistics for the years 2008–2010. Author Amsterdam (2013).

[47] Cayotte E Buchow H. Who dies of what in Europe before the age of 65. Eurostat Statistics in Focus 67/2009 (2009).

[48] Steinhagen-Thiessen E Borchelt M. Morbidity, medication, and functional limitations in very old age. Berlin Aging Study Aging From. (1999) 70:131–66. 10.1017/CBO9780511586545.006

[49] Henchoz K Cavalli S Girardin M. Health perception and health status in advanced old age: a paradox of association. J Aging Stud. (2008) 22:282–90. 10.1016/j.jaging.2007.03.00229998108

[50] Johnson RJ Wolinsky FD. The structure of health status among older adults: disease, disability, functional limitation, and perceived health. J Health Soc Behav. (1993). 34:105–21. 10.2307/2137238 8277124

[51] Bostan C Oberhauser C Stucki G Bickenbach J Cieza A. Biological health or lived health: which predicts self-reported general health better? BMC Public Health. (2014) 14:189. 10.1186/1471-2458-14-189 24555764 PMC3943451

[52] Bonner WIA Weiler R Orisatoki R Lu X Andkhoie M Ramsay D. Determinants of self-perceived health for Canadians aged 40 and older and policy implications. Int J Equity Health. (2017) 16:94. 10.1186/s12939-017-0595-x 28587654 PMC5461772

[53] Fors S Lennartsson C Lundberg O. Health inequalities among older adults in Sweden 1991–2002. Eur J Public Health. (2007) 18:138–43. 10.1093/eurpub/ckm097 17925323

[54] Suresh S Sabanayagam C Shankar A. Socioeconomic status, self-rated health, and mortality in a multiethnic sample of US adults. J Epidemiol. (2011) 21:337–45. 10.2188/jea.JE20100142 21747210 PMC3899432

[55] OECD/European Observatory on Health Systems and Policies. Portugal: Country Health Profile 2017, State of Health in the EU, OECD Publishing, Paris/European Observatory on Health Systems and Policies, Brussels (2017). 10.1787/9789264283527-en

[56] Eikemo TA Bambra C Judge K Ringdal K. Welfare state regimes and differences in self-perceived health in Europe: a multilevel analysis. Soc Sci Med. (2008) 66:2281–95. 10.1016/j.socscimed.2008.01.022 18314241

[57] Kunst AE Bos V Lahelma E Bartley M Lissau I Regidor E. Trends in socioeconomic inequalities in self-assessed health in 10 European countries. Int J Epidemiol. (2004) 34:295–305. 10.1093/ije/dyh342 15563586

[58] Subramanian S Kawachi I. Being well and doing well: on the importance of income for health. Int J Soc Welfare. (2006) 15:S13–22. 10.1111/j.1468-2397.2006.00440.x30212153

[59] Grundy E Sloggett A. Health inequalities in the older population: the role of personal capital, social resources and socio-economic circumstances. Soc Sci Med. (2003) 56:935–47. 10.1016/S0277-9536(02)00093-X 12593868

[60] Hoeymans N Feskens EJ Kromhout D Van den Bos GA. The contribution of chronic conditions and disabilities to poor self-rated health in elderly men. J Gerontol Series A Biomed Sci Med Sci. (1999) 54:M501–6. 10.1093/gerona/54.10.M501 10568532

[61] Arokiasamy P Uttamacharya Jain K. Multi-morbidity, functional limitations, and self-rated health among older adults in India: cross-sectional analysis of LASI pilot survey, 2010. Sage Open. (2015) 5:2158244015571640. 10.1177/2158244015571640

[62] Uutela T Kautiainen H Järvenpää S Hakala M Häkkinen A. Self-rated health in patients with rheumatoid arthritis is associated with health-related quality of life but not with clinical variables. Scand J Rheumatol. (2016) 45:288–93. 10.3109/03009742.2015.1116604 26807489

[63] Bindman AB Grumbach K Osmond D Komaromy M Vranizan K Lurie N. Preventable hospitalizations and access to health care. JAMA. (1995) 274:305–11. 10.1001/jama.274.4.305 7609259

[64] Syed ST Gerber BS Sharp LK. Traveling towards disease: transportation barriers to health care access. J Community Health. (2013) 38:976–93. 10.1007/s10900-013-9681-1 23543372 PMC4265215

[65] Wong SY-s Chung RY-n Chan D Chung GK-k Li J Mak D. What are the financial barriers to medical care among the poor, the sick and the disabled in the Special Administrative Region of China? PLoS ONE. (2018) 13:e0205794. 10.1371/journal.pone.0205794 30427845 PMC6235271

[66] Parikh PB Yang J Leigh S Dorjee K Parikh R Sakellarios N. The impact of financial barriers on access to care, quality of care and vascular morbidity among patients with diabetes and coronary heart disease. J Gen Intern Med. (2014) 29:76–81. 10.1007/s11606-013-2635-6 24078406 PMC3889957

[67] Huot S Ho H Ko A Lam S Tactay P MacLachlan J. Identifying barriers to healthcare delivery and access in the Circumpolar North: important insights for health professionals. Int J Circumpolar Health. (2019) 78:1571385. 10.1080/22423982.2019.1571385 30696379 PMC6352934

[68] Kyriopoulos I-I Zavras D Skroumpelos A Mylona K Athanasakis K Kyriopoulos J. Barriers in access to healthcare services for chronic patients in times of austerity: an empirical approach in Greece. Int J Equity Health. (2014) 13:54. 10.1186/1475-9276-13-54 25062725 PMC4113665

[69] Carmo Id Dos Santos O Camolas J Vieira J Carreira M Medina L. Prevalence of obesity in Portugal. Obesity Rev. (2006) 7:233–7. 10.1111/j.1467-789X.2006.00243.x 16866971

[70] Marques-Vidal P Dias CM. Trends and determinants of alcohol consumption in Portugal: results from the national health surveys 1995 to 1996 and 1998 to 1999. Alcoholism Clin Exp Res. (2005) 29:89–97. 10.1097/01.ALC.0000150001.31722.D1 15654297

[71] Tremblay S Dahinten S Kohen D. Factors related to adolescents' self-perceived health. Health Rep. (2003) 14(Supp.):7–16. 14768290

[72] Buneviciene I Bunevicius R Bagdonas S Bunevicius A. The impact of pre-existing conditions and perceived health status on mental health during the COVID-19 pandemic. J Public Health. (2022) 44:e88–95. 10.1093/pubmed/fdab248 34179996

[73] Dalmases M Benítez I Sapiña-Beltran E Garcia-Codina O Medina-Bustos A Escarrabill J. Impact of sleep health on self-perceived health status. Sci Rep. (2019) 9:1–7. 10.1038/s41598-019-43873-5 31086269 PMC6513841

[74] Margaretten M Julian L Katz P Yelin E. Depression in patients with rheumatoid arthritis: description, causes and mechanisms. Int J Clin Rheumatol. (2011) 6:617. 10.2217/ijr.11.62 22211138 PMC3247620

[75] Pinquart M Shen Y. Depressive symptoms in children and adolescents with chronic physical illness: an updated meta-analysis. J Pediatr Psychol. (2011) 36:375–84. 10.1093/jpepsy/jsq104 21088072

[76] Mulsant BH Ganguli M Seaberg EC. The relationship between self-rated health and depressive symptoms in an epidemiological sample of community-dwelling older adults. J Am Geriatr Soc. (1997) 45:954–8. 10.1111/j.1532-5415.1997.tb02966.x 9256848

[77] Pinquart M Sörensen S. Influences of socioeconomic status, social network, and competence on subjective well-being in later life: a meta-analysis. Psychol Aging. (2000) 15:187. 10.1037/0882-7974.15.2.187 10879576

[78] Brummett BH Barefoot JC Siegler IC Clapp-Channing NE Lytle BL Bosworth HB. Characteristics of socially isolated patients with coronary artery disease who are at elevated risk for mortality. Psychosom Med. (2001) 63:267–72. 10.1097/00006842-200103000-00010 11292274

[79] Cornwell EY Waite LJ. Social disconnectedness, perceived isolation, and health among older adults. J Health Soc Behav. (2009) 50:31–48. 10.1177/002214650905000103 19413133 PMC2756979

[80] Berkman LF Glass T Brissette I Seeman TE. From social integration to health: Durkheim in the new millennium. Soc Sci Med. (2000) 51:843–57. 10.1016/S0277-9536(00)00065-4 10972429

[81] Cornman JC Goldman N Glei DA Weinstein M Chang M-C. Social ties and perceived support: two dimensions of social relationships and health among the elderly in Taiwan. J Aging Health. (2003) 15:616–44. 10.1177/0898264303256215 14587529

[82] Ahn S Basu R Smith ML Jiang L Lorig K Whitelaw N. The impact of chronic disease self-management programs: healthcare savings through a community-based intervention. BMC Public Health. (2013) 13:1141. 10.1186/1471-2458-13-1141 24314032 PMC3878965

[83] Shaaban AN Morais S Peleteiro B. Healthcare Services utilization among migrants in portugal: results from the National Health Survey 2014. J Immigrant Minority Health. (2018). 21:1–11. 10.1007/s10903-018-0744-3 29644552

[84] Perelman J Chaves P de Almeida JMC Matias MA. Reforming the Portuguese mental health system: an incentive-based approach. Int J Mental Health Syst. (2018) 12:25. 10.1186/s13033-018-0204-4 29853991 PMC5975562

[85] de Almeida JC. Portuguese national mental health plan (2007–2016) executive summary. Mental Health Family Med. (2009) 6:233–44. PMC2873880 22477915

